# integrating single-cell transcriptomics and chromatin accessibility profiling to decipher the molecular landscape of Hepatocellular carcinoma

**DOI:** 10.1371/journal.pone.0359329

**Published:** 2026-10-15

**Authors:** Kaile Pan, Lijian Chen, Yitao Zheng

**Affiliations:** Department of General Surgery, Ningbo No. 2 Hospital, Wenzhou Medical University, Ningbo, Zhejiang, China; Versiti Blood Research Institute, UNITED STATES OF AMERICA

## Abstract

**Background:**

Hepatocellular carcinoma (HCC) is a prevalent and lethal liver malignancy, characterized by high recurrence rates and limited therapeutic options. Current diagnostic and treatment strategies are hindered by drug resistance and a lack of effective biomarkers for early detection.

**Methods:**

This study aims to elucidate the role of lactylation and fibroblast cells within the tumor microenvironment (TME) of HCC, focusing on their potential as therapeutic targets. Utilizing a comprehensive multi-omics approach, we integrated single-cell RNA sequencing (scRNA-seq) and single-cell ATAC sequencing (scATAC-seq) data from public databases (GEO, TCGA) to explore transcriptional and epigenetic landscapes at single-cell resolution.

**Results:**

Our analysis identified fibroblast cells as key players with high lactylation-related pathway activity, and CREB1 as a central transcription factor regulating lactylation-associated genes in these cells. The CREB1 regulatory network was associated with specific immune cell infiltration patterns, drug sensitivity profiles, and enrichment in critical signaling pathways such as Chemokine, TNF, NF-κB, and EMT. These findings underscore the pivotal role of lactylation-driven epigenetic regulation in fibroblasts, influencing the HCC immune microenvironment and disease progression.

## 1 Introduction

Hepatocellular carcinoma (HCC) represents the most prevalent form of primary liver cancer and remains a significant global health challenge, with a particularly high incidence in East Asia and Sub-Saharan Africa, where it is closely linked to endemic hepatitis B virus (HBV) infection and dietary exposure to aflatoxin [1,2]. The pathogenesis of HCC is a complex, multi-step process that typically arises from a background of chronic liver injury and subsequent regeneration. This progressive deterioration of hepatic tissue is primarily driven by etiological factors such as chronic viral hepatitis (HBV and hepatitis C virus), alcohol-related liver disease, and the emerging epidemic of metabolic dysfunction-associated fatty liver disease [3–5]. At the cellular level, the persistent cycle of hepatocyte damage and regeneration creates a mutagenic environment, fostering the accumulation of genetic mutations and epigenetic alterations that ultimately drive malignant transformation [6,7]. This pathological process is further exacerbated by disruptions in hepatic metabolism, which contribute to a pro-carcinogenic milieu characterized by inflammation and fibrosis. Key molecular mechanisms underpinning HCC development include the inactivation of tumor suppressor genes such as TP53 and the aberrant activation of oncogenic signaling pathways, most notably the Wnt/β-catenin cascade [8–10]. Consequently, a deeper understanding of the intricate etiology of HCC and the urgent need to identify novel and reliable biomarkers are paramount for improving early detection, therapeutic strategies, and the overall dismal prognosis associated with this malignancy.

Despite significant advances in understanding hepatocarcinogenesis, the clinical management of HCC remains challenging and requires comprehensive evaluation of tumor stage, hepatic functional reserve, and overall patient performance status. For patients diagnosed at early stages, curative-intent therapeutic options include surgical resection, liver transplantation, and locoregional ablative therapies; among these, surgical resection and liver transplantation represent the optimal treatment strategies, with transplant recipients meeting Milan criteria achieving favorable 5-year survival rates of approximately 70% [11–13]. However, the majority of patients present with intermediate to advanced disease at diagnosis, for whom transarterial chemoembolization (TACE) serves as the standard locoregional intervention, while systemic therapy constitutes the mainstay of treatment. Current systemic regimens encompass molecular targeted agents such as sorafenib and lenvatinib, as well as combination immunotherapy approaches, notably atezolizumab plus bevacizumab, which have demonstrated improved survival outcomes compared to monotherapy [14–17]. Post-treatment surveillance protocols emphasize regular cross-sectional imaging and serum alpha-fetoprotein monitoring, with particular attention to ongoing antiviral therapy and metabolic management in patients with underlying cirrhosis. Nevertheless, the prognosis for advanced HCC remains dismal, with median overall survival limited to 1–2 years and 5-year survival rates persistently below 20% [18,19]. These sobering statistics underscore that early detection and multimodal therapeutic integration are critical to improving clinical outcomes, while the continued development of novel targeted agents and immunotherapeutic strategies remains an active area of clinical investigation.

In recent years, bioinformatics analysis has been extensively applied to microarray datasets, enabling the systematic identification of pivotal genes and facilitating subsequent functional investigations in cancer biology. The advent of next-generation sequencing technologies has revolutionized our understanding of tumor heterogeneity, particularly through the emergence of single-cell RNA sequencing (scRNA-seq) and single-cell assay for transposase-accessible chromatin using sequencing (scATAC-seq). These cutting-edge approaches provide unprecedented resolution in dissecting intercellular heterogeneity, thereby illuminating the intricate relationships between cellular diversity and tumor progression, metastatic potential, and therapeutic resistance [20,21]. By integrating bulk transcriptomic data with single-cell multi-omics profiling, researchers can now delineate complex regulatory networks and identify master regulators that drive malignant transformation. Accordingly, the present study aims to integrate transcriptomic datasets, scRNA-seq, and scATAC-seq data to systematically identify and functionally characterize key transcription factors involved in hepatocarcinogenesis, thereby providing novel insights into the molecular mechanisms underlying HCC and potentially uncovering promising therapeutic targets.

## 2 Materials and methods

### 2.1 Data acquisition

1) GEO database (https://www.ncbi.nlm.nih.gov/geo/info/datasets.html) is the full name of GENE EXPRESSION OMNIBUS, which is a gene expression database created and maintained by the National Center for Biotechnology Information (NCBI) of the United States. 46 complete single-cell expression spectrum disease sample data of GSE151530 were downloaded from the NCBI GEO public database for single-cell transcriptome analysis. 16 scATAC-seq data of GSE227265 were downloaded from the NCBI GEO public database for scATAC-seq analysis. The lactylation-related gene set used in this study was derived from a previously established and validated 23‑gene signature reported by Huang et al [22], which encompasses genes involved in lactate metabolism, histone lactylation, and downstream signaling pathways.
2) The Cancer Genome Atlas(TCGA) database (https://portal.gdc.cancer.gov/) is currently the largest cancer gene information database, which stores data including gene expression data, copy number variation, SNP, etc. The mRNA expression data of 424 patients with hepatocellular carcinoma (TCGA-LIHC) were included from the TCGA public database, including 50 normal cases and 374 tumor cases. Our analysis used publicly available summary statistics. No new data were collected, and no new ethical approval was required.

### 2.2 Quality control

First, the expression profile is read in through the Seurat package, where we filter cells based on the total number of Unique Molecular Identifier(UMI) per cell, the number of genes expressed, and the mitochondrial expression ratio and ribosomal expression ratio of each cell. The expression ratio of mitochondrial genes and ribosomal genes refers to the percentage of the total expression of mitochondrial genes and ribosomal genes in the total expression of all genes. Cells with high mitochondrial gene expression ratio and ribosomal gene ratio have low RNA expression, and these cells are entering the death program. We perform quality control through the median absolute deviation (MAD), and data with a median absolute deviation of more than 3 times is generally considered to be an outlier. So far, cell quality control is completed.

### 2.3 Data standardization and cell annotation

We used the global normalization LogNormalize method to adjust the total expression of each cell to 10000 by multiplying a coefficient s0, and then took the logarithm for normalization; used CellCycleScoring to calculate the cell cycle score; FindVariableFeatures to find highly variable genes; we used the ScaleData function to remove the gene expression fluctuations caused by the expression proportion of mitochondrial genes, the expression proportion of ribosomal genes and the different cycles of cells; RunPCA performed linear dimensionality reduction on the expression matrix and selected the principal components for subsequent analysis; Harmony was used to remove batch effects, and RunUMAP unified manifold approximation and projection (UMAP) was used for nonlinear dimensionality reduction. Clusters were annotated through literature and CellMarker databases, and were annotated to some cells that are important to the disease. SingleR software automated annotation was used to find the cell types and corresponding marker genes in the corresponding tissues for cell annotation.

### 2.4 Ligand-receptor interaction analysis (CellChat)

CellChat is a tool that can quantitatively infer and analyze intercellular communication networks from single-cell data. CellChat uses network analysis and pattern recognition methods to predict the main signal inputs and outputs of cells, as well as how these cells and signals coordinate functions. In this analysis, we used the standardized single-cell expression profile as input data, and the cell subtypes obtained from single-cell analysis as cell information. We analyzed cell-related interactions and quantified the closeness of the interaction relationship by the strength (weigths) and number (count) of the interaction between cells, so as to observe the activity and influence of each type of cell in the disease. For CellChat analysis, cell‑cell interactions were considered significant when the permutation‑based p‑value was below 0.01, and the number of permutations was set to 1000.

### 2.5 SCENIC analysis

SCENIC (single-cell regulatory network inference and clustering) is a method based on co-expression and motif analysis to calculate the reconstruction of gene regulatory networks and cell state identification of single-cell transcriptome data. It first identifies the gene set co-expressed with transcription factors through GENIE3, then performs motif enrichment analysis on each co-expression module, retains significantly enriched motifs, and uses the database to annotate the motifs with Transcription Factor(TF). The annotation results are divided into high and low confidence. TFs directly annotated by the database and inferred by homologous genes are high-confidence results, and TFs annotated using motif sequence similarity are low-confidence results. The genes in the co-expression module are scored with the retained motif, and genes with significantly high scores are identified (understood as motifs being very close to the TSS of these genes). The genes in the co-expression module with low scores in the motif are deleted, and the remaining gene set is called a regulatory unit (regulon). Each regulon is a gene set of a transcription factor and its directly regulated target genes. The next work of SCENIC is to score the activity of each regulon in each cell. The basis of the score is the expression value of the gene. The higher the score, the higher the activation degree of the gene set. The obtained activity matrix can identify the cell type and state. The regulon specificity score (RSS) finds the regulon specific to the cell subset. The function calcRSS in the R package SCENIC is used to complete it. The RSS values of different regulons can be directly compared in the same cell type, while the RSS values of the same regulon cannot be directly compared between different cell types. When comparing the specificity of the same regulon between different cell types, the RSS can also be converted to a z-score to reflect which cell type the regulon is more specific to. Different regulons are converted into z-scores by data conversion, which reflects which cell type the regulon is more specific to. For SCENIC analysis, genes expressed in at least 5% of cells with a minimum expression level of 0.1 were retained. Dimensionality reduction was performed using 30 principal components for both t‑SNE and UMAP, and regulons were identified with an adjusted p‑value < 0.05.

### 2.6 scATAC-seq data processing

scATAC-seq was analyzed using the Signac (v1.10.0) R package. Low-quality cells were removed according to the following criteria: nCount_peaks > 200, nCount_peaks < 20000, TSS.enrichment < 8, blacklist_fraction < 0.01, nucleosome_signal < 3, TSS.enrichment > 1.75. Peak region fragments represents the total number of fragments in the peak, reads in peaks represents the ratio of fragments in the scATAC-Seq peak, blacklist ratio represents the ratio of reads in the genomic blacklist region defined by ENCODE, nucleosome signal represents the nucleosome signal intensity, and TSS enrichment represents the TSS enrichment score of the transcription start site defined by ENCODE. The term frequency-inverse document frequency (TF-IDF) was then used for normalization, and all peaks were selected as variable features for LSI dimensionality reduction and analysis. The Run UMAP function in the Seurat package was used for unified manifold approximation and projection (UMAP) nonlinear dimensionality reduction. Finally, the gene activity was calculated using the GeneActivity function, and the gene activity matrix was added to the Seurat object as a new detection using the CreateAssayObject function.

### 2.7 Integrated analysis of scRNA-seq and scATAC-seq data

To help interpret scATAC-seq data, we applied Seurat’s integrated framework to identify corresponding cell pairs between the two modal data. The shared correlation patterns between scATAC-seq gene activity and scRNA-seq gene expression were determined by the FindTransferAnchors function (reduction = ‘cca’). The cell type label of each cell in the scATAC-seq data was then predicted by the TransferData function (weight.reduction = ‘lsi’ and dim = 2:30). For scATAC‑seq data, peaks were identified with MACS2 (q‑value < 0.05), and enriched motifs were determined at an FDR < 0.05.

### 2.8 Identification of specific regulatory elements

To investigate differences in chromatin accessibility between key cell types, we first identified differential peaks in scATAC-seq data using MACS223, which were defined as differentially chromatin regions (DARs) (p_val_adj <  0.05 and log2(fold change (FC)) > 1). To identify genomic regions where peaks were identified, such as promoter regions, introns, and exons, peak annotation was performed using the annotatePeak function in the ChIPSeeker R package. Promoter regions were designated as −3,000 bp to +3,000 bp of TSS. The transcript-related object used for genomic region mapping was TxDb.Hsapiens.UCSC.hg38.knownGene.

### 2.9 Single-cell transcription factor activity analysis

To quantify the enrichment of TF motifs, we used chromVAR built into the Signac (v1.10.0) R package. First, using the merged cell type specific peak set from scATAC-seq, we added motif information from the JASPAR2020 core vertebrate set to the peaks. After adding the motif information, we used the RunChromVAR function in Signac to calculate the chromVAR motif deviation score. Using the chromVAR deviation matrix, we used the FindMarkers function to calculate the differentially enriched chromVAR motifs.

### 2.10 Immune Infiltration

The CIBERSORT method is a widely used evaluation method for immune cell types in microenvironments. This method is based on the principle of support vector regression and performs deconvolution analysis on the expression matrix of immune cell subtypes. It contains 547 biomarkers that distinguish 22 human immune cell phenotypes, including T cells, B cells, plasma cells, and myeloid cell subsets. This study used the CIBERSORT algorithm to analyze patient data to infer the relative proportions of 22 immune infiltrating cells and to perform correlation analysis between gene expression and immune cell content.

### 2.11 Drug sensitivity analysis

Based on the largest pharmacogenomics database (GDSC Cancer Drug Sensitivity Genomics Database, https://www.cancerrxgene.org/), we used the R package “oncoPredict” to predict the chemotherapy sensitivity of each tumor sample; the IC50 estimate for each specific chemotherapy drug treatment was obtained by regression method, and the regression and prediction accuracy were tested by 10 cross-validation tests using the Genomics of Drug Sensitivity in Cancer (GDSC) training set. All parameters selected the default values, including “combat” to remove batch effects and taking the average of repeated gene expression.

### 2.12 GSEA analysis

According to the expression of key genes of patients, patients were divided into high and low expression groups, and the differences in signal pathways between the two groups were further analyzed by Gene Set Enrichment Analysis (GSEA). The background gene set is the version 7.0 annotation gene set downloaded from the MsigDB database as the annotation gene set of the subtype pathway, and the differential expression analysis of the pathways between different groups was performed, and the significantly enriched gene sets (adjusted p value less than 0.05) were ranked according to the consistency score. GSEA analysis is often used to explore the close combination of disease typing and biological significance.

### 2.13 GSVA analysis

Gene set variation analysis (GSVA) is a non-parametric and unsupervised method for evaluating transcriptome gene set enrichment. GSVA converts gene-level changes into pathway-level changes by comprehensively scoring the gene set of interest, thereby determining the biological function of the sample. This study will download gene sets from the Molecular signatures database, use the GSVA algorithm to comprehensively score each gene set, and evaluate the potential biological function changes of different samples.

### 2.14. Statistical analysis

All statistical analyses were performed using R language (version 4.3.2), and p < 0.05 was considered statistically significant.

## 3 Results

### 3.1 Preliminary processing of single-cell expression profile data

This analysis uses expression profiles containing 46 liver cancer-related tissue samples. First, the expression profile is read in through the Seurat package, where we filter cells based on the total number of UMIs per cell, the number of genes expressed, and the percentage of mitochondrial readings per cell. It is generally believed that if a variable is more than 3 MAD away from the median, the value is considered an outlier and needs to be eliminated. A total of 43,561 cells, filtered violin plots and scatter plots (S1A-S1B Fig). The 10 genes with the highest standard deviation are displayed (S1C Fig).

### 3.2 Single-cell data cell subpopulation annotation

The optimal number of PCs was observed through ElbowPlot: 20 (S1D Fig); PCA dimensionality reduction analysis found that there was a batch effect between samples, and further Harmony analysis was used to reduce the dimensionality and remove the batch (S1E Fig), and finally 12 subtypes were obtained through UMAP. This study further annotated each subtype, and 12 clusters were annotated to 10 cell categories, including T cells, B cells, Plamas cells, Macrophages, Plasmacytoid dendritic cells, Fibroblast cells, Endothelial cells, Hepatocytes, Cholangiocytes, and Cyclings (Fig 1A), bubble diagrams of classic markers of 10 types of cells (Fig 1B), and cell proportion bar graphs (Fig 1C). Then FindAllMarker was used to find differential genes in cell subpopulations, and the top six genes with the largest logFC among differential genes were proposed. ClusterGVis and org.Mm.e.g.,db were called for enrichment analysis, and visCluster was used to visualize the heat map (Fig 1D).

**Fig 1:**
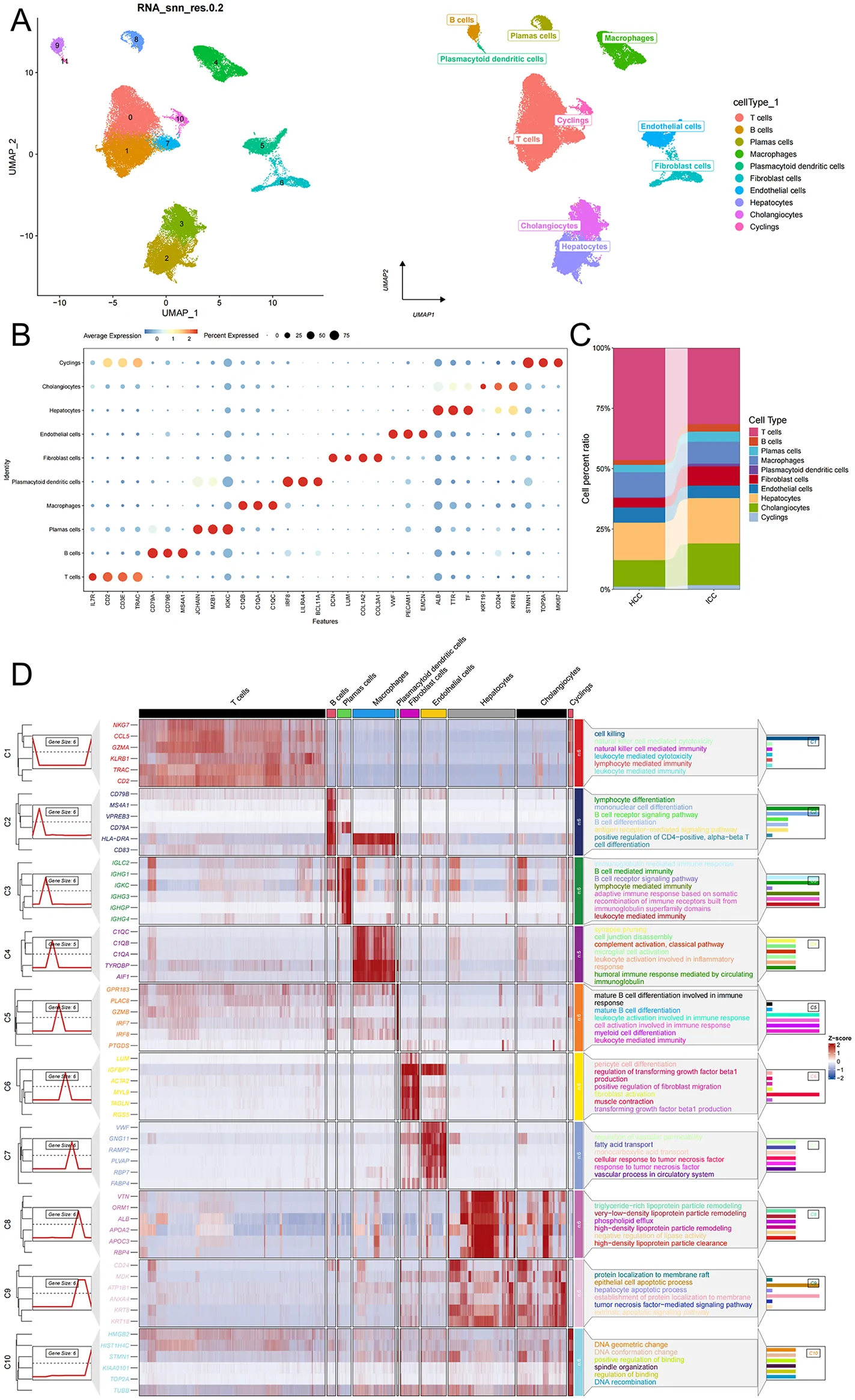
Cell type annotation and related analysis of single-cell RNA sequencing (scRNA-seq) data. A. UMAP clustering of single cells with a clustering resolution. Cells were classified and annotated into ten cell types with different colors. B. Bubble plot showing the average expression (color) and expression positive rate (bubble size) of canonical marker genes in each cell subtype. C. Stacked bar graph presenting the percentage distribution of each cell subpopulation. D. Enrichment analysis of six differential genes.

### 3.3 Ligand-receptor interaction analysis (Cellchat) and multi-algorithm quantification of key pathway scores and differential analysis of high and low scores

We used the software package cellchat to analyze the ligand-receptor relationship of the features in the single-cell expression profiles of these two groups. We found that there are complex interaction relationships between these cell subtypes (Fig 2A). To further explore intercellular signal transduction and show the ligands of Fibroblast cells and other cells (Fig 2B). Then we obtained the lactylation-related gene set from the literature [22]. We used AUCell, UCell, singscore, ssGSEA and Add algorithms at the scRNA-seq level to evaluate the expression of different cell types by averaging the scores of the above algorithms, and took the cell subtype with the largest score as the key cell. Among them, Fibroblast cells have the highest score in multiple quantitative key pathways, so Fibroblast cells are taken as key cells. Extract the high and low score groups of Fibroblast cells for differential analysis (Fig 2C).

**Fig 2:**
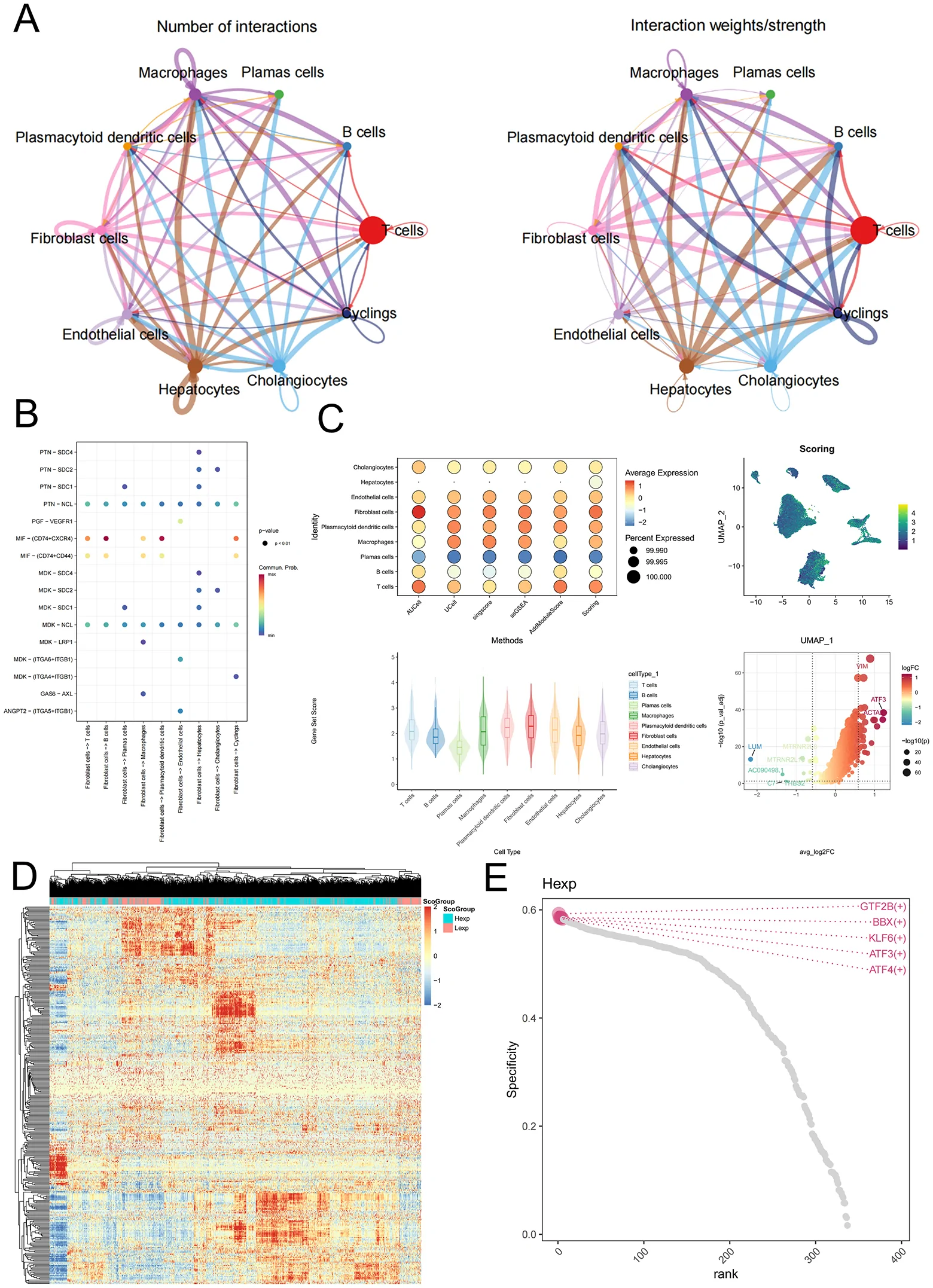
Analysis of intercellular communication, cell type annotation, and gene expression characteristics in single-cell RNA sequencing (scRNA-seq) data. A. CellChat intercellular communication network. Left: total number of ligand-receptor interactions; Right: interaction strength between cell subtypes. Colored lines represent cell-cell signaling. B. Dot plot showing fibroblast-derived ligand-receptor pairs. Dot color indicates P-value, dot size represents communication probability. C. Comprehensive validation of annotated cell subtypes, including marker gene bubble plot, expression violin plot, score UMAP distribution and volcano plot. D. Lactylation score heatmap E. Analysis of lactylation characteristic transcription factor scatter plot and display of the top 5 transcription factors.

### 3.4 SCENIC analysis

We selected the Fibroblast cell subpopulation for SCENIC analysis and drew a heat map to visualize the regulon activity score of the high lactylation group (Fig 2D). We also use scatter plots to show the specific ranking of transcription factors in the high lactylation group, the specific ranking of each group of regulon, and the top 5 targets (Fig 2E). The horizontal axis represents the ranking and the vertical axis represents the RSS score. The regulator with higher RSS may be specifically related to the cell population. All TFs were then extracted as candidate TFs (scRNA_TF.txt).

### 3.5 scATAC-seq data processing and integration of scRNA and scATAC

The DensityScatter function was used to visualize the relationship between variables stored in the object metadata and quickly find the appropriate cutoff value for different QC indicators (Fig 3A). The DepthCor function was used to determine the correlation between each LSI component and sequencing depth (Fig 3B). After quality control, a total of 21,967 cells were retained in the scATAC-seq data for subsequent analysis (Fig 3C). We then calculated the prediction score using Seurat’s label transfer algorithm and identified 9 cell types in the scATAC-seq dataset that were the same as scRNA-seq (Fig 3D). By integrating scRNA-seq and scATAC-seq multimodally (Fig 3E), and using genes used for cell annotation in scRNA-seq, we can visualize the open DA peaks in 9 cells, close to some key marker genes of these cell types such as C1QA. Here, we will highlight the DA peak area in gray (Fig 3F).

**Fig 3:**
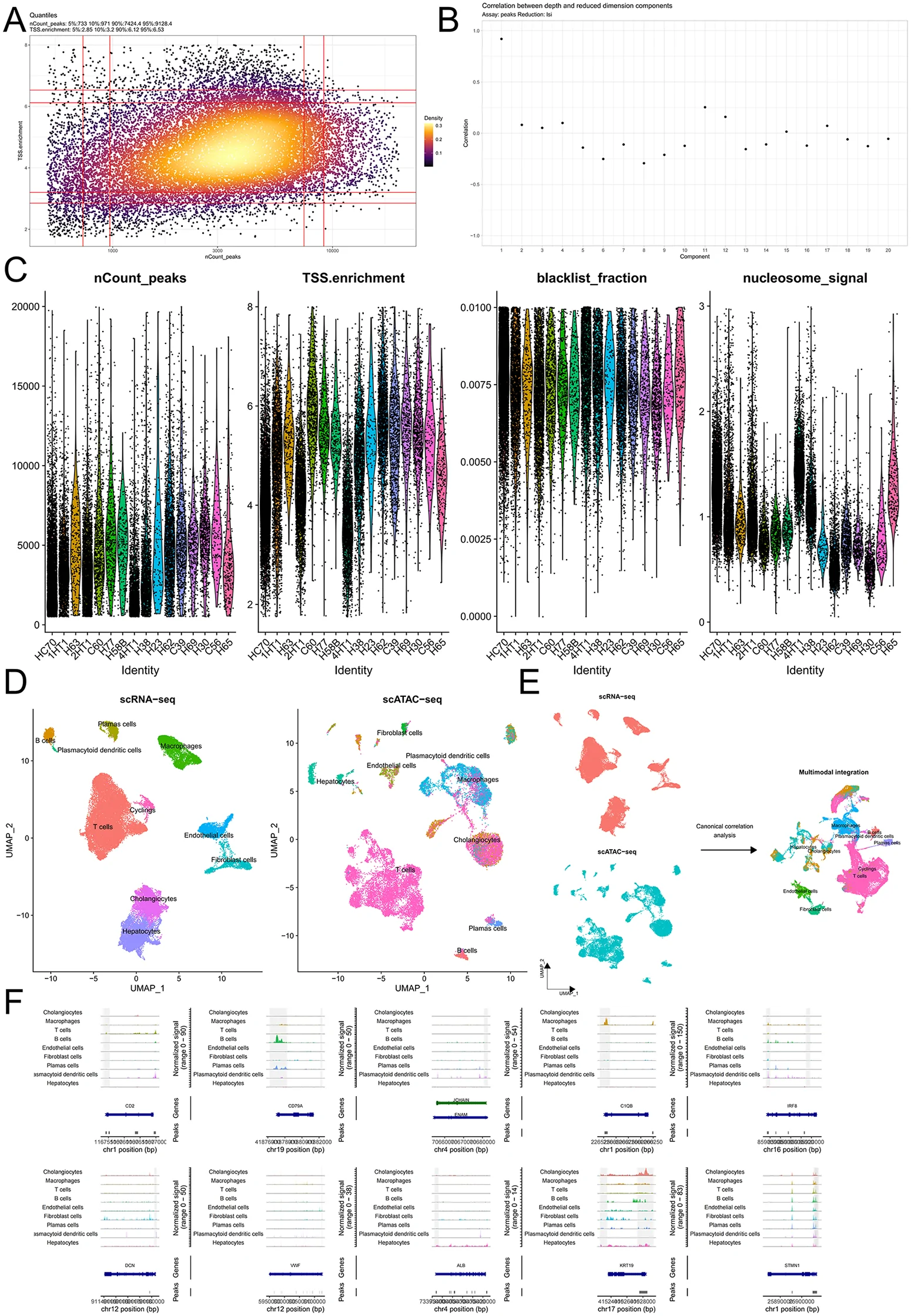
Integration of single-cell ATAC sequencing data analysis. A. Scatter density plot of cell quality in single-cell ATAC-seq data B. Correlation analysis between sequencing depth and dimensionality reduction components C. Violin plot of quality control metrics for each sample D. Comparison of cell type annotations between scRNA-seq and scATAC-seq E. Multimodal integration analysis of scRNA-seq and scATAC-seq F. Visualization results of integrated analysis of single-cell multi-omics (RNA+ATAC) data.

### 3.6 Identification of specific regulatory elements and determination of key genes

Use Seurat’s FindAllMarker function to find scATAC-seq differential peaks, and the screening conditions are (avg_log2FC > 1, p_val_adjl < 0.05, cellType = Fibroblast cells) to draw a heat map of the Z-score normalized accessibility of scATAC-seq differential peaks of key cell types (Fig 4A). We used the ChIPSeeker R package to annotate the obtained differential peaks, and the results showed that most of the differential peaks were located in the promoter, intron and other regions of the genome (Fig 4B). Then, the differential peaks were enriched, and the results showed that GO was enriched in pathways such as Extracellular Matrix Organization, Renal System Development, and Cell−Substrate Adhesion, and KEGG was enriched in pathways such as Focal Adhesion and Rap1 Signaling Pathway (Fig 4C-4D).

**Fig 4:**
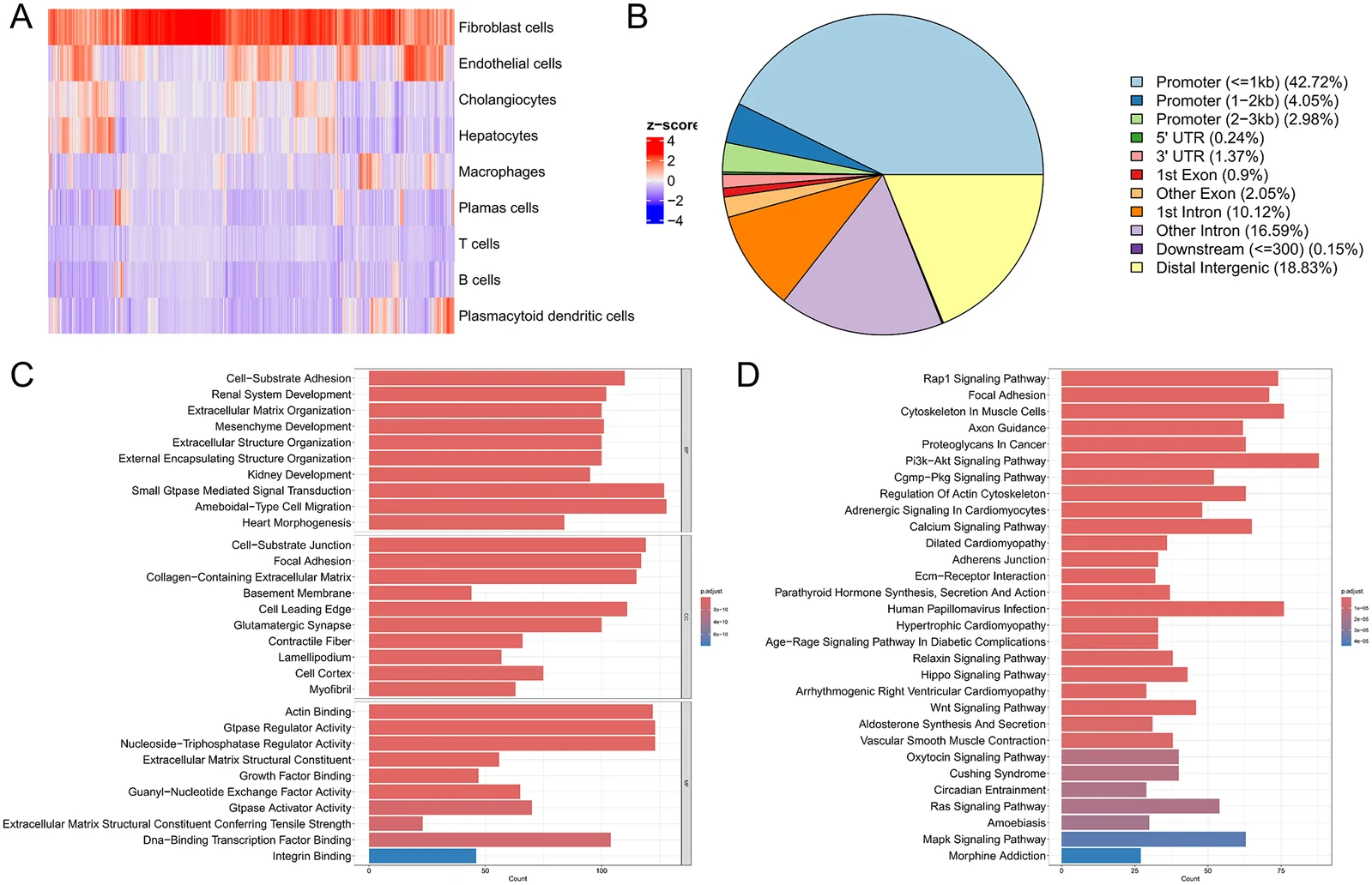
Differential analysis and enrichment analysis. A. scATAC-seq chromatin accessibility heatmap across annotated cell subtypes. Color gradient (red to blue) represents high to low z-score of peak accessibility. B. Peak accessibility profiles and genomic functional region distribution of cell type-specific marker genes C. Bar plot showing top enriched Gene Ontology (GO) terms of differential accessible peaks. Bar length corresponds to enrichment score. D. Bar plot displaying top enriched KEGG signaling pathways of differential accessible peaks. Bar length indicates enrichment magnitude.

### 3.7 Transcription factor activity analysis and footprint analysis

In this study, AddMotif was called to add DNA sequence motif information, the differential peaks of Fibroblast cells in the previous step were called, and FindMotif was called to find motifs that were over-represented in the given genomic feature set of differential peaks. We found 150 motif TFs (Enriched.motifs.csv) that act specifically on differential peaks, with the screening conditions of fold.enrichment>1 and p.adjust<0.05. We intersected the motif TFs and the TFs in Scenic to obtain 74 TFs (Fig 5A). We then quantified lactic acid-related genes in the TCGA transcriptome expression profile, obtained lactic acid scores, and made correlation scatter plots with the 74 TFs. The correlation coefficient filtering condition was 0.3, and the p value was 0.05. The most relevant TF was CREB1. Therefore, CREB1 is the key regulator for our subsequent analysis. Then we showed the sequence logo of the key regulator, showing the preferred sequence of the transcription factor at the DNA binding site (Fig 5B). Then, footprint analysis was used to show the Tn5 insertion enrichment of the key regulator in different groups. The Tn5 insertion enrichment showed similar distribution characteristics near the binding site (Fig 5C).

**Fig 5:**
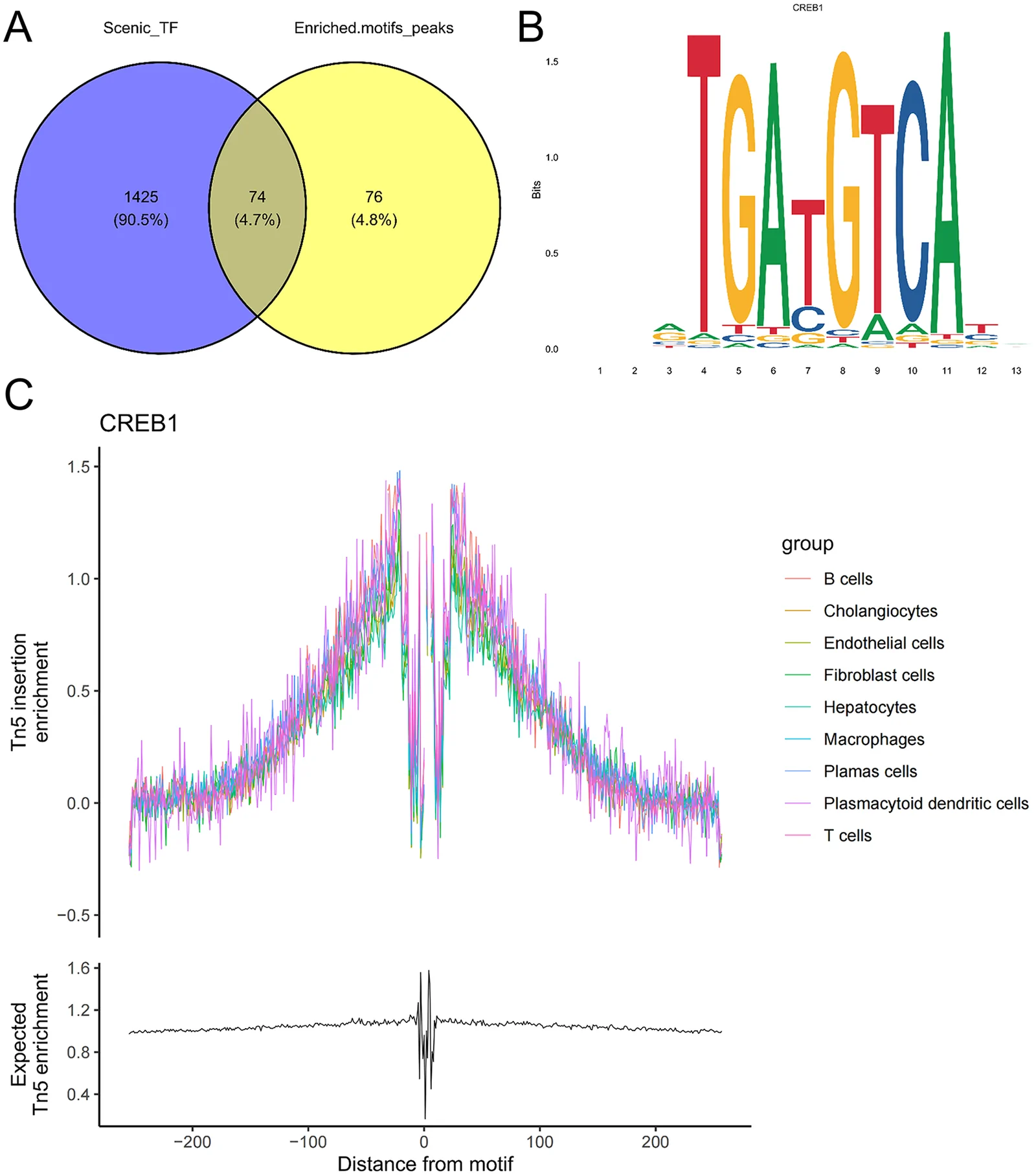
Identification of key transcription factors (TF). A. Venn diagram showing the intersection of specific TFs in differential peaks with differential peaks in Fibroblast cells B. Sequence logo of transcription factor CREB1 C. Footprint analysis of CREB1 transcription factor (TF) reveals its binding activity in various cell types.

### 3.8 Immune infiltration

Then, the target gene corresponding to CREB1 was obtained in Scenic, and TFs were quantified using ssgsea in the TCGA transcriptome expression profile to obtain CREB1_moderator. The microenvironment is mainly composed of fibroblasts, immune cells, extracellular matrix, multiple growth factors, inflammatory factors and special physical and chemical characteristics. The microenvironment significantly affects the diagnosis, survival outcome and clinical treatment sensitivity of the disease. We showed the distribution of immune infiltration levels and the correlation with immune cells in different forms (Fig 6A-6B). Compared with the control group, the levels of immune cells such as Macrophages M0 and Dendritic cells resting in the disease group samples were upregulated (Fig 6C). We further explored the relationship between key genes and immune cells and found that CREB1_Moderator was significantly positively correlated with B cells naive, Plasma cells, T cells CD4 memory resting, and Dendritic cells resting, and significantly negatively correlated with T cells CD4 naive, T cells follicular helper, T cells gamma delta, NK cells resting, NK cells activated, Macrophages M0, Dendritic cells activated, and Mast cells resting (Fig 6D). In addition, we also analyzed the correlation between key regulators and various immune factors, including immunosuppressive factors, immunostimulatory factors, chemokines, and receptors. These analyses indicate a potential correlation between key regulators and immune cell infiltration levels, and suggest a possible involvement of these regulators in the immune microenvironment (Fig 6E-6I).

**Fig 6:**
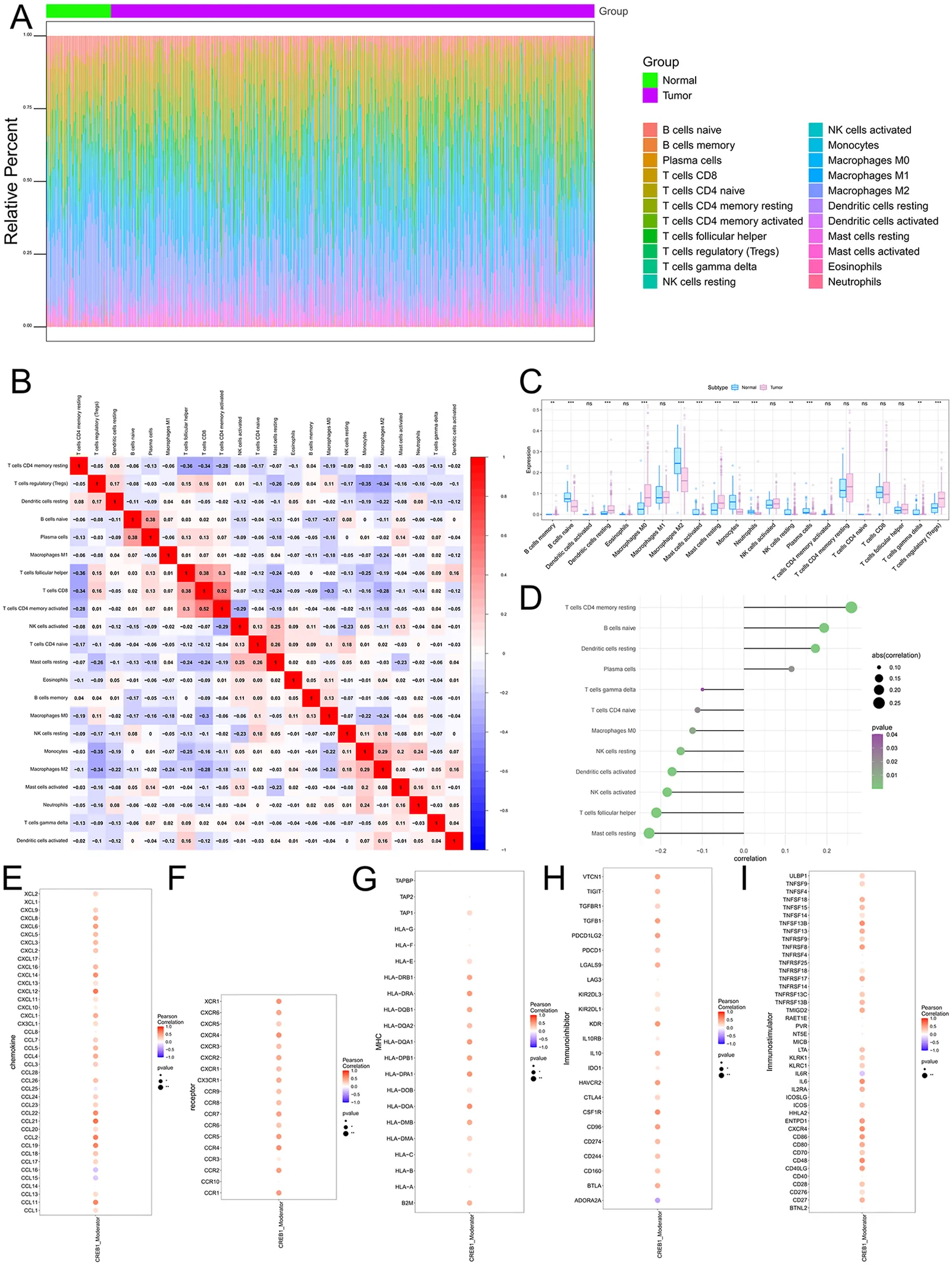
Immune infiltration landscape of CREB1. A. Comparison of Immune Cell Composition between Normal Tissue and Tumor Tissue. B. Correlation between Immune Infiltration Level and Immune Cells. C. Analysis of Differences in Immune Cell Proportions between Normal Tissue and Tumor Tissue. D. Correlation Analysis between Immune Cell Infiltration and Patient Prognosis. E-I. Correlation between CREB1 and Immunosuppressive Factors, Immunostimulatory Factors, Chemokines, and Receptors.

### 3.9 Relationship between key regulators and drug sensitivity

Our study is based on the drug sensitivity data of the GDSC database. The R package “oncoPredict” is used to predict the chemotherapy sensitivity of each tumor sample and explore the sensitivity of CREB1_Moderator to common chemotherapy drugs. The results showed that CREB1_Moderator was significantly correlated with Camptothecin_1003, Vinblastine_1004, Cisplatin_1005, Cytarabine_1006, Gefitinib_1010, and MK-2206_1053 (Fig 7A). **It should be emphasized that the drug sensitivity predictions derived from the GDSC and oncoPredict algorithms are computational estimates and have not been experimentally verified. Therefore, these results should be interpreted with caution and serve as a basis for future in vitro or in vivo validation.**

**Fig 7:**
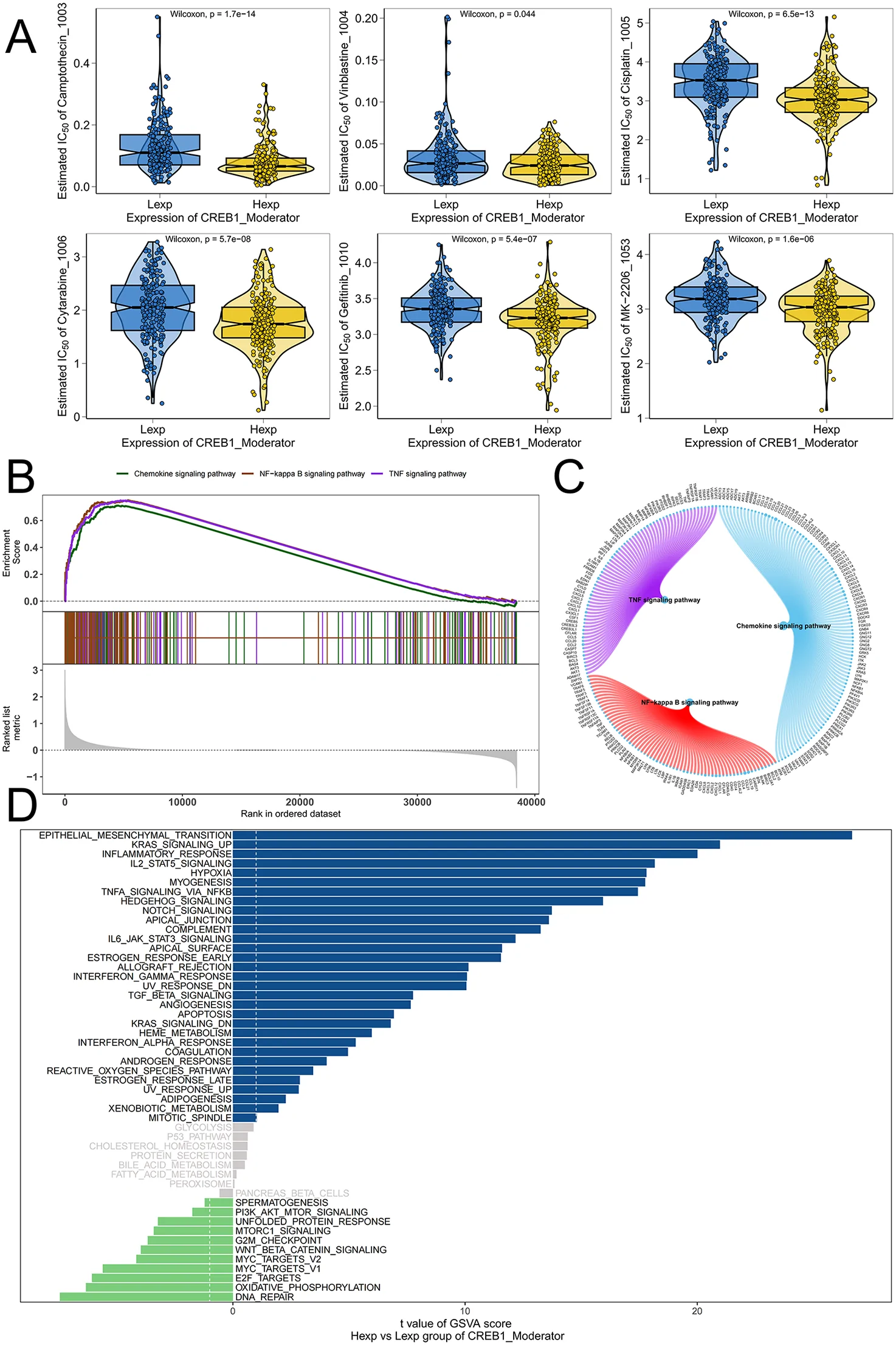
Biological function analysis of CREB1. A. Analysis of CREB1 sensitivity to conventional chemotherapeutic drugs B-C. Gene set enrichment analysis (GSEA) reveals differences in molecular characteristics of CREB1 D. Bar chart of differentially activated Hallmark pathways between high CREB1 expression group and low CREB1 expression group.

### 3.10 Signaling pathways involved in key regulators

Next, we studied the specific signaling pathways involved in key genes and explored the potential molecular mechanisms by which key genes affect disease progression. GSEA results showed that CREB1_moderator was enriched in signaling pathways such as Chemokine signaling pathway, TNF signaling pathway, and NF-kappa B signaling pathway (Fig 7B-7C). GSVA analysis showed that CREB1_moderator was enriched in signaling pathways such as EPITHELIAL_MESENCHYMAL_TRANSITION and KRAS_SIGNALING_UP (Fig 7D). This suggests that key genes may affect disease progression through these pathways.

### 3.11 Relationship between key regulators and disease-related genes

We obtained disease-related genes through the GeneCards database (https://www.genecards.org/). The relationship between key regulators and disease-related genes was explored through correlation analysis. This study analyzed the expression levels of 50 genes with high Relevance*score rankings and expressed in the transcriptome. The correlation coefficient filtering condition was 0.2, and the p value was 0.05. Correlation coefficients greater than 0.2 were significantly positively correlated, and those less than −0.2 were significantly negatively correlated. The correlation scatter plot between key regulators and genes related to disease regulation was shown (Fig 8).

**Fig 8:**
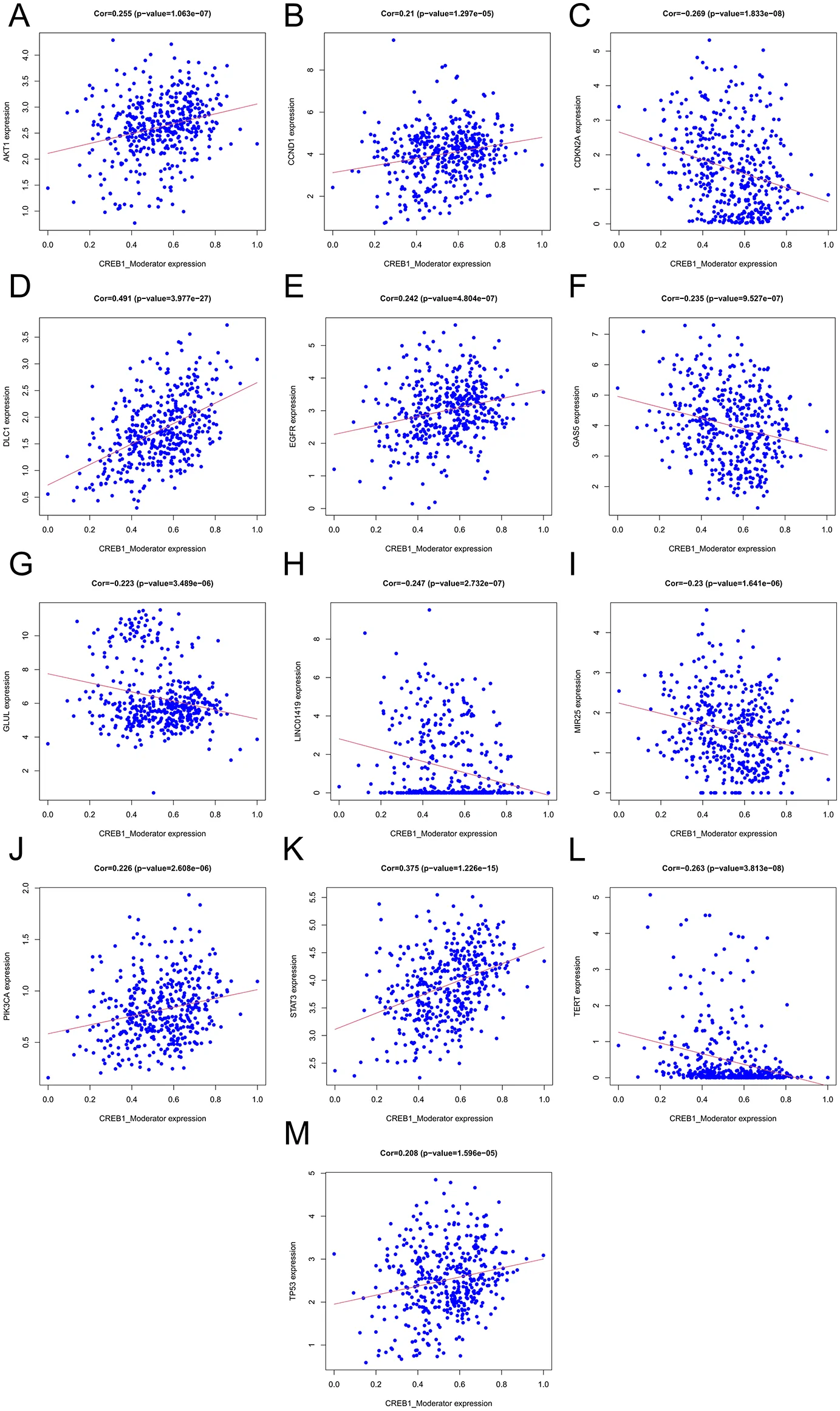
Scatter plot of correlation analysis between CREB1 expression and key regulatory genes.

### 3.12 Immune metabolic pathways

We used AUCell to quantify the scores of target genes in single cells according to the target genes corresponding to key regulators, and obtained the CREB1_Moderator key regulator. AUCell was then used to quantify and score genes in immune metabolism-related pathways in single cells, and a bubble chart was used to display the activity differences of key genes in immune metabolism-related pathways. The results showed that CREB1_Moderator was more active in pathways such as tgf_beta_signaling (S2 Fig).

## 4 Discussion

In this study, we conducted a comprehensive integrative analysis of transcriptomic data from hepatocellular carcinoma (HCC) patients in the TCGA database, integratedwith single-cell RNA sequencing (scRNA-seq) and single-cell assay for transposase-accessible chromatin sequencing (scATAC-seq) datasets from the GEO repository. Our study leveraged the high cellular resolution and in-depth profiling capacity of scRNA-seq and scATAC-seq, the complementary and mutually validating strengths greatly facilitated the identification of key transcription factors and subsequent functional annotations in HCC. Through this multi-omics approach, we identified CREB1 as a critical lactylation-associated transcription factor that may serve as a promising prognostic biomarker and potential therapeutic target in HCC.

The current therapeutic landscape for hepatocellular carcinoma encompasses surgical resection, liver transplantation, chemoradiotherapy, and targeted therapies based on tyrosine kinase inhibitors [23–27]. In recent years, immune checkpoint inhibitors targeting the PD-1/PD-L1 axis have achieved substantial advancements and demonstrated promising efficacy across various malignancies [28–30]. However, HCC is characterized by a profoundly immunosuppressive tumor microenvironment, in which malignant cells evade immune surveillance through diverse mechanisms, including aberrant activation of the Wnt/β-catenin signaling pathway, p53 mutations, and epigenetic silencing of immune-related genes [31–35]. Notably, Li Q et al. reported that HCC cells express PD-L1 to bind to PD-1 receptors on T cells, thereby suppressing T-cell activity and facilitating immune escape [36]. Furthermore, the tumor immune microenvironment is populated by immunosuppressive cell populations, such as tumor-associated macrophages (TAMs), regulatory T cells (Tregs), and cancer-associated fibroblasts (CAFs), which collectively promote tumor progression and metastasis [37–39]. Consequently, disrupting this immunosuppressive milieu represents a critical therapeutic imperative for HCC management. Our findings lay the foundation for such strategies, offering novel insights into the diagnosis, treatment, and prognostic stratification of hepatocellular carcinoma.

Recent rapid advances in bioinformatics and molecular research have provided numerous novel insights into the epigenetic regulation and pathogenic mechanisms of hepatocellular carcinoma [40–44]. By integrating single-cell RNA sequencing with single-cell ATAC sequencing, we identified CREB1 as a lactylation-associated key transcription factor in HCC and conducted comprehensive analyses of its immune infiltration landscape, drug sensitivity, and related signaling pathways. The transcription factor cAMP response element-binding protein 1 (CREB1) belongs to a subclass of the basic leucine zipper (bZIP) superfamily and possesses the potential to regulate approximately 4,000 genes [45]. As a proto-oncogene, CREB1 overexpression has been implicated in various malignancies [46]. CREB1 exerts its transcriptional regulatory functions through homodimerization or heterodimerization and by binding to cis-regulatory elements [47]. Furthermore, CREB1 can modulate the expression of growth factor-related genes and can be activated through multiple pathways involving cAMP and calcium influx [48,49]. Lei Zhu et al. demonstrated that CREB1-mediated lipogenesis promotes the progression of hepatocellular carcinoma [50]. Additionally, CREB1 phosphorylation has been reported to induce drug resistance in prostate cancer [51] and to enhance tumor cell proliferation in colon cancer [52,53]. Liang X et al. verified the prominent pro-tumorigenic effects of the PKA/CREB signaling axis in hepatocellular carcinoma (HCC) via a combination of in vitro cellular assays and in vivo animal experiments [54]. Given that the oncogenic roles of CREB1 have been extensively documented across multiple malignancies, including HCC, our study enriches the understanding of CREB1 in hepatocellular carcinoma through the integration of multi-omics datasets.

In summary, our study characterizes the bioinformatic profile of CREB1 in hepatocellular carcinoma (HCC), including its correlation with HCC-related gene expression, regulatory involvement in key signaling pathways, and potential molecular mechanisms underlying its oncogenic role. By integrating multi-omics data, we present a comprehensive characterization of CREB1 as a lactylation-related transcription factor with prognostic and therapeutic relevance. However, several limitations warrant acknowledged. First, our findings are primarily based on public databases, which may introduce inherent biases related to sample selection, platform heterogeneity, and batch effects. Second, while our analyses suggest functional implications of CREB1 in HCC, the associated biological roles and regulatory mechanisms require further experimental validation through in vitro and in vivo studies; Third, a limitation of this study is that the drug sensitivity profiles were inferred from publicly available pharmacogenomic databases (GDSC) via the oncoPredict algorithm, which relies on gene expression data and machine‑learning models. These in silico predictions, while informative, do not substitute for functional experiments such as cell viability assays or patient‑derived organoid tests. Thus, the current results should be viewed as hypothesis‑generating, and further experimental validation is essential to substantiate the identified associations. Despite these limitations, our work provides a foundational framework for understanding CREB1-mediated regulation in HCC and highlights its potential as a novel therapeutic target. This study may ultimately contribute to the development of more precise and effective combination strategies for HCC treatment.

## Supporting information

S1 Fig Single-cell RNA sequencing data quality control. A. Violin plot for evaluating the sequencing data quality of different samples. B. Scatter plot of single-cell data. C. Analyzed highly mutated genes and displayed the top 10 genes. D. ElbowPlot for principal component analysis. E. PCA principal component analysis.
(TIF)

S2 Fig Bubble plot of immunometabolism-related pathways. (TIF)

S1 Data Supporting data files. CREB1_Moderator.cor.xls: correlation analysis results for CREB1 moderators; Enriched.motifs.csv: enrichment analysis results of transcription factor binding motifs; GO.txt: Gene Ontology (GO) enrichment analysis results; KEGGId.txt: KEGG pathway enrichment analysis results and related pathway IDs; Lactylation.cor.xls: correlation analysis results for lactylation; scRNA_TF.txt: transcription factor analysis results from single-cell RNA sequencing.
(ZIP)
